# Brief Report: Attentional Cueing to Images of Social Interactions is Automatic for Neurotypical Individuals But Not Those with ASC

**DOI:** 10.1007/s10803-018-3592-z

**Published:** 2018-04-25

**Authors:** Marcus Neil Morrisey, Catherine L. Reed, Daniel N. McIntosh, M. D. Rutherford

**Affiliations:** 1 Department of Psychology, Neuroscience and Behaviour, McMaster University, 1280 Main Street West, Hamilton, ON L8S 4K1 Canada; 2 Department of Psychology, Claremont McKenna College, Claremont, USA; 3 Department of Psychology, University of Denver, Denver, USA

**Keywords:** Autism spectrum disorders, Social cognition, Social orienting, Reflexive attention

## Abstract

Human actions induce attentional orienting toward the target of the action. We examined the influence of action cueing in social (man throwing toward a human) and non-social (man throwing toward a tree) contexts in observers with and without autism spectrum condition (ASC). Results suggested that a social interaction enhanced the cueing effect for neurotypical participants. Participants with ASC did not benefit from non-predictive cues and were slower in social contexts, although they benefitted from reliably predictive cues. Social orienting appears to be automatic in the context of an implied social interaction for neurotypical observers, but not those with ASC. Neurotypical participants’ behavior may be driven by automatic processing, while participants with ASC use an alternative, effortful strategy.

## Introduction

Autism spectrum condition (ASC) is a spectrum of neurodevelopmental anomalies characterized by impaired social cognition and communication, and circumscribed interests or rigid adherence to routine (American Psychiatric Association 2013). Although social cognitive deficits are broadly understood to characterize ASC, attentional orienting to social stimuli is usually tested in this population using isolated socially relevant cues to direct attention, like faces (Dawson et al. 2005; Koldewyn et al. 2013; Walsh et al. 2014) or eye gaze (Landry and Parker 2013; Nation and Penny 2008; Pruett et al. 2011). Outside of the laboratory, social cues typically occur in a context that includes other people or objects. When the sociality of the stimuli involves an implied dyadic or triadic social interaction, ASC-related anomalies in visual processing of human bodies may be more apparent. The current study addresses this question by comparing the attentional effects of a directional action on observers with and without ASC in the context of social and non-social actions.

### Social Orienting and Joint Attention

Social orienting is a rapid, involuntary, attentional shift towards a social cue. An example is an attentional shift in the direction of another’s eye gaze (Frischen et al. 2007). This occurs in observers without ASC, or neurotypical (NT) observers even when the direction of the gaze is not predictive of the target location (Friesen and Kingstone 1998) or reliably points in the direction opposite the target (Hill et al. 2010). Similar attentional cueing occurs with non-social directional cues like arrows (Ristic et al. 2002; Tipples 2002), but evidence indicates these non-social cueing effects are qualitatively different from those triggered by social cues (Marotta et al. 2012), are not effective when the cue is counter-predictive (Friesen et al. 2004; but see Tippler 2008 for a counter-example), and involve different neural mechanisms than the response to eye-gaze (Akiyama et al. 2006).

The speed advantage measured on trials where the location of the target is c orrectly cued, compared to trials where the opposite side of the display is cued is called the validity effect. Landry and Parker (2013) reported a meta-analysis that validity effects with eye-gaze stimuli are the same among NT observers and those with ASC, and the most reliable group effect is that those with ASC show slower response times on all trial types. These two groups are equally sensitive to the predictiveness (contingency) of the cue. The cuing effect with eye-gaze stimuli is stronger than with arrow stimuli, and this difference is stronger for ASC than NT control observers (Landry and Parker 2013). Another review suggests that validity effects are attenuated in ASC but also notes that methodological differences have led to varied results (Sacrey et al. 2014). Even though a lack of gaze-following in naturalistic settings is considered an early diagnostic symptom of ASC (Lord et al. 1989) individuals with ASC have been found to orient to eye gaze in laboratory settings (Chawarska et al. 2003; Rutherford and Krysko 2008; Swettenham et al. 2003).

Importantly, most of the above studies involve stimuli that portray eye gaze without a socially relevant target. It is possible that differences between NT observers and those with ASC would emerge if the display involved a triadic social interaction. Bayliss and Tipper (2005) used an orienting task with central social (face with gaze to the left and right) and non-social (arrows) cues with flanking social (faces) and non-social (scrambled faces, tools) objects. NT individuals who showed fewer symptoms of autism on the Autism-Spectrum Quotient (ASQ) scale (Baron-Cohen et al. 2001), showed stronger cue effects when a social interaction was implied by a gaze towards another face than for other trial types. This enhanced cuing was not observed in participants who showed more symptoms of autism on the ASQ (Bayliss and Tipper 2005).

### Social Orienting and Implied Action

Viewing a human in a pose that implies action can trigger social orienting. For example, when a central human figure appears poised to throw a ball, attention follows the implied trajectory of the ball (Gervais et al. 2010). Gaze, gesture, and head position cues are also integrated automatically to predict the direction of a person’s attention (Langton and Bruce 2000).

### Perception of Bodies

In typical perception, people show a greater inversion effect for social stimuli such as faces (e.g. Yin 1969) and bodies (Reed et al. 2003) compared to non-social stimuli. This means that there is a greater perceptual cost to inverting social stimuli, and it is taken as evidence for specialized perceptual processing of social stimuli. Those with ASC show an attenuated inversion effect when making same/different judgments comparing two sequential body postures, suggesting less specialized processing of bodies than is seen among NT observers (Reed et al. 2007). For NT individuals, attentional orienting occurs in response to eye direction (Friesen and Kingstone 1998; Ristic et al. 2005) and body direction in the absence of flanking images (Gervais et al. 2010), in a social context (Bristow et al. 2007), or when embedded in a non-social context, such as when flanked by power tools (Bayliss and Tipper 2005). The current study was designed to test the effect of a social context, indicated by flankers, on attentional orienting responses in an implied directional action display, testing NT viewers and those with and without ASC.

### The Current Study

This study investigates three related questions: (1) Does the social context, here the sociality of the target of implied human action, affect orienting responses triggered by such cues? (2) Is such attentional orienting automatic? (3) Do observers with and without ASC differ in their response to the implied social context?

To examine how a social (a human figure) versus a non-social (a tree) recipient of the implied action was would affect attentional responses, we used spatial attentional cueing elicited by body posture. We modified Gervais et al.’s (2010) covert orienting paradigm: a central photograph showed a man poised to throw a ball either to the left or right. Targets appeared on the side consistent (valid cue) or inconsistent (invalid cue) with the throw’s direction. We modified the stimuli by adding flanking images so that we could manipulate the sociality of the context: the recipient of the implied action was either a man standing where he could receive the throw (social interaction) or a tree standing where it could be hit by the throw (non-social interaction). The human recipient is inherently directional, facing the central thrower, while the non-social tree stimulus is symmetrical about the vertical axis. This difference enhances the sense of interaction with the social stimulus, in contrast with the non-social stimulus. Nonetheless, both flanker images depicted a meaningful functional context for the action depicted by the cue: a person can throw a ball to a person or at a tree.

The first hypothesis is that the social context (human vs. tree flanker) would affect the orienting response, measured as reaction time (RT) to target detection. Although we expect responses to be faster for valid cues than invalid cues overall, the social meaning of throwing a ball to a human should facilitate valid trials (i.e., the target appears in the same direction that the center figure throws the ball) in the social more than in the non-social trials. If a human is throwing a ball to a human flanker, a social interaction is implied whereas if he is throwing a ball at a tree, there is no social interaction implied. Specifically, if the flankers are human, then RTs should be relatively facilitated for validly cued trials when the target appears on the side the central figure is facing compared to invalidly cued trials, compared to this same interaction if the flankers are trees. This context difference should occur regardless of cue predictability.

The second hypothesis, the automaticity of attentional shifts, was assessed in two ways. First, we created predictive and non-predictive trials: the direction of the central figure was either associated with the location of target or it was not. Participants saw only one of these two trial types. If the attentional response is automatic, we would expect to see cuing even in non-predictive conditions, because in the absence of a reliable relationship between the direction of the central cue and location of the target, any RT advantage in the direction of the central image is attributed to the inherent attentional directing of the cue. Therefore, this contrast between predictive and non-predictive cue conditions will be used to assess whether the attentional shift in response to the central figure is automatic. Second, we created short (150 ms) and long (300 ms) stimulus onset asynchronies (SOAs) between the cue and target. Reflex-like attentional shifts are thought to occur with SOAs of 150 ms or less for lateralized cues; cognitively mediated attention shifts are thought to occur with SOAs above 300 ms (Posner and Cohen 1984). A cuing effect at longer SOAs would be consistent with more volitional (as opposed to automatic) processing.

The third hypothesis is that there will be social orienting differences between ASC and NT groups. Specifically, the difference in context (between human and tree flanker trials) is expected to be smaller for the ASC group than for the NT group. In addition, the automaticity of social orienting (human flanker trials) predicted by the second hypothesis is expected to be less apparent in the ASC group than in the NT group.

Finally, we tested whether IQ interacts with our hypotheses and findings. Other studies have indicated that IQ may have differential influences on performance for those with ASC but not NT. For example, perception of biological motion in point light displays, is correlated with intelligence in ASC but not NT participants (Rutherford and Troje 2011). Also, it been shown that FSIQ scores for individuals with ASC affect communication more than social skills (Black et al. 2009).

## Method

### Participants

Twenty-five neurotypical (“NT”) participants (six female, mean age 30.4 years) were recruited through online advertisement. Twenty-three participants (four female; mean age 29.1 years) who had a clinical diagnosis of ASC and met ADOS-G (mean ADOS-G score 13.46, SD 4.97; Lord et al. 2000) criteria were recruited through a local residential facility. This was our original stopping criteria for recruitment, as it constitutes the total number of participants with ASC in this age group who were available to participate in this study. Participants were paid $10.00 per hour.

As an inclusion criteria, all participants reported normal or corrected-to-normal vision. Participants with a FSIQ below 70 (two NT), or who responded to more than eight catch trials or failed to respond to more than eight experimental trials (one NT, two ASC) were excluded from the study. Two additional participants (one NT, one ASC) were identified as overly influential data points and removed (see “Results” section) leaving a total of 21 NT and 20 individuals with ASC included in the analyses. Equivalence testing (Kirkwood and Westlake 1981) confirmed that NT and ASC groups were matched on age, full-scale IQ, performance IQ, and verbal IQ. These measures did not differ significantly among four groups: participants with ASC in the predictive and the non-predictive conditions and NT participants in the predictive and the non-predictive conditions (See Table 1).

**Table 1:** Group demographics and equivalence testing

	Age	WAIS		
		VIQ	PIQ	FSIQ
NT				
Mean	31	96.2	100.2	98.7
SD	8.9	12.3	16.3	12.8
Range	20–50	70–117	70–125	73–118
ASD				
Mean	29.1	96.8	97.7	95.6
SD	8.9	12.9	14.5	11.6
Range	19–58	75–113	69–138	77–117
95% CI for equivalence of means	(− 6.3, 2.58)	(− 6.26, 6.44)	(− 10.8, 4.7)	(− 9.3, 3)
95% CI for ratio of variances	(0.4, 2.29)	(0.45, 2.61)	(0.32, 1.88)	(0.33, 1.94)

### Procedure

Stimuli were presented using a laptop PC with a 16:9 aspect ratio LCD screen and E-Prime 2.0 software (Psychology Software Tools, Pittsburgh, PA). Participants were tested individually either in the laboratory or a quiet room at a residential facility. During the two-hour session, participants completed the consent form, heard instructions, completed the computer task, and, if not on file, completed the Weschler Adult Intelligence Scales (Wechsler 1997).

Participants viewed the screen from 60 cm away using a fixed chin rest. Trials were blocked into social and non-social context blocks: In social context blocks, flanker images depicted men facing inwards; in non-social blocks the images depicted trees. Throughout the trials two, identical flanking photographic images of either men or trees (both were 1.5**°** × 7.2**°**) were visible on the left and right sides of the screen against a white background; their centers were 5**°** from the center of the screen. During the inter-trial interval (ITI), the central ‘+’ was also visible.

**Fig. 1:**
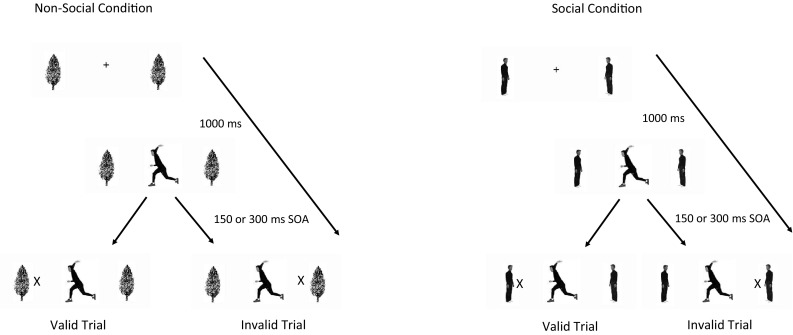
A schematic of an experimental trial

On each trial (Fig. 1), participants fixated on a central black ‘+’(1.4**°** × 1.4**°**) on a white background. It was replaced by a central image of a Caucasian, dark-haired, male dressed in black clothes (3.4**°** × 7.2**°**) posed as if throwing a ball, facing either to the left or right. After a stimulus onset asynchony (SOA) of 150 or 300 ms, a target ‘X’ (1.4**°** × 1.4**°**) appeared between one flanking image and the central cue. The target appeared either on the same side the thrower was facing (valid cue) or behind the thrower (invalid cue). The display remained until participants detected the target and pressed the spacebar or 2000 ms had elapsed. Participants were instructed to respond as quickly as possible without making errors. The ITI duration was randomly chosen from five values spaced evenly across the interval 1000–3000 ms.

Participants were randomly assigned to either predictive or non-predictive cue conditions, each with 10% catch trials (no target, no response). In predictive cue conditions, 70% of trials were valid (targets appeared on the same side the cue was facing), and 20% were invalid (targets appeared on the opposite side the cue was facing). In non-predictive cue conditions, 45% were valid and 45% were invalid. Participants were not given any information about the predictiveness of the cue. After 12 practice trials, participants completed one social context and one non-social context block with 44 trials each, for a total of 88 experimental trials. Order was counterbalanced across participants.

## Results

### Preliminary Treatments

RTs that were less than 200 ms or greater than 3 SD above their group’s mean and non-responses were excluded from the analysis (3% of trials). RTs were inverse transformed to better fit the normality assumption (Maxwell and Delaney 2004). Linear models were constructed following techniques laid out by Laird and Ware (1982), and calculated using the NLME package (Pinheiro et al. 2014) in the R statistical package (R Core Team 2015). Follow up pairwise t-tests were adjusted for multiple comparisons using the Holm–Bonferroni method.

**Fig. 2:**
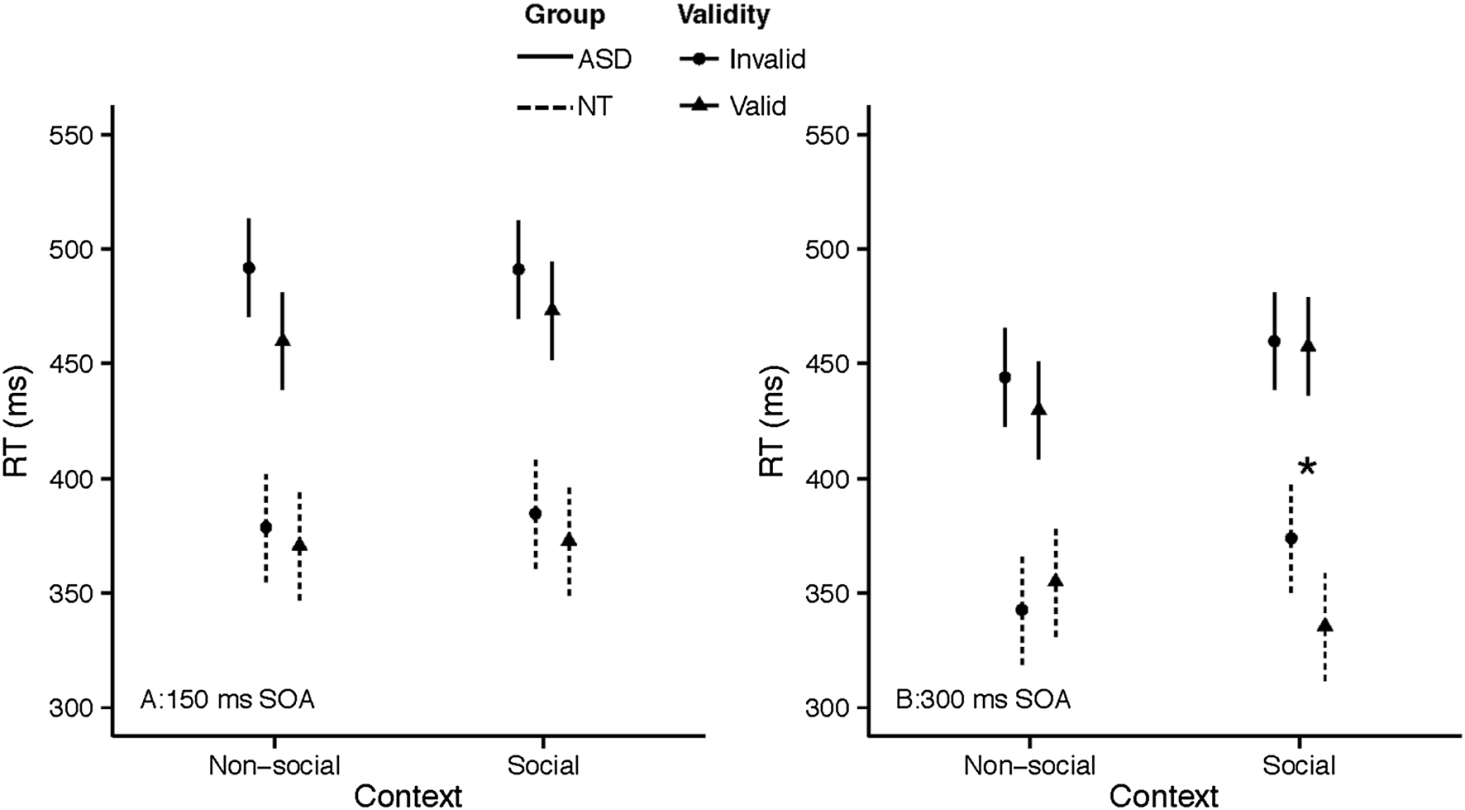
Reaction time by group, context, validity, & SOA. Error bars represent 1 SE

We fit a 2-level hierarchical linear model with random intercepts using RT as our dependent variable. The first level included all main effects and interactions of the between-participant variables: group (2: ASC, NT), cue condition (2: predictive, non-predictive), and FSIQ as well as the repeated measures factors: context (2: social, non-social), validity (2: valid cue, invalid cue), and SOA (2: 150, 300 ms) which were nested within the higher order effect (i.e., random effect) of participants. Model diagnostics and residual plots suggested that the model was appropriate for the data with no significant outliers in the fixed or random effects. Nonetheless, when fitting hierarchical models containing a small number of higher-level terms (i.e., participants) with a large number of observations, highly influential higher-level effects, i.e., participants with highly aberrant scores, can unduly influence the regression model and may not show up as outliers in the data (Van der Meer et al. 2010). Therefore, we used the influence.ME package (Nieuwenhuis et al. 2012) to iteratively drop each participant from the model and refit it to examine the influence on model parameters using three measures: Cook’s Distances, DFBETAs, and percentile change. One participant was removed from each group for exceeding accepted cutoffs on all three measures (as mentioned in the “Participants” subsection of the “Methods” section). Therefore, analyses reported here included data from 21 NT and 20 ASC participants. RTs are graphed by condition in Fig. 2.

Statistically significant effects and interactions are displayed in Table 2.

**Table 2:** Statistically significant effects and interactions of the full model

Term	*F*	*df*	Residual df	*p*
Group	12.9	1	33	0.001
Validity	31.8	1	3101.04	< 0.001
SOA	137.1	1	3101.02	< 0.001
Group:context	8	1	3101.04	0.005
FSIQ:context	7.2	1	3101.01	0.007
Cue:validity	8.1	1	3101.05	0.005
FSIQ:SOA	8.5	1	3101.01	0.004
Group:context:validity	4.2	1	3101.05	0.04
FSIQ:context:validity	5	1	3101.02	0.026
Group:cue:FSIQ:context	9.1	1	3101.01	0.003
Cue:FSIQ:validity:SOA	5.8	1	3101.01	0.016

As expected from the covert orienting paradigm, there were main effects of Validity and SOA. We measured validity effects by subtracting invalid from valid trials. We found a significant validity effect (i.e., RTs to validly cued trials were faster than to invalidly cued trials); participants were faster if the central figure faced towards rather than away from the target (valid trials: *M* = 435, *SE* = 25; invalid trials: *M* = 459, *SE* = 25). Also, longer SOAs produced faster responses (300 ms SOA: *M* = 431, *SE* = 25; 150 ms SOA: *M* = 462, *SE* = 25).

The first hypothesis was that the sociality of the context, i.e., presence of a social recipient (human vs. tree flankers) for a throw, would have an effect on RT, such that the validity effect would be stronger for social contexts over non-social contexts, regardless of cue predictability. We found a significant Group × Context × Validity interaction (see Table 2). Inspection of Fig. 2 reveals that NT participants performed faster in the social context. However, participants with ASD showed the opposite pattern. Further, for the NT group, the validity of the trial lead to faster RTs, but only for the valid trials. This interaction supports the first hypothesis and the third hypothesis, that there would be group differences in the effect of social context.

To simplify and examine the a four-way cue condition × FSIQ × Validity × SOA interaction as well as the other higher-order interactions, we split the model by Cue Condition into predictive and non-predictive models but retained all other variables.

### Non-predictive Cue Condition

All significant effects and interactions of the non-predictive Cue Condition model appear in Table 3. We measured validity effects by subtracting invalid from valid trials. The presence of a significantly negative difference, therefore, indicates an RT advantage when the target appears in the cued direction. The validity main effect confirmed a significant cueing advantage for both Groups across Contexts and at both SOAs; (*valid*–*invalid* = − 23 ms, *SE* = 10 ms). An SOA main effect indicated that both Groups were faster to respond when the SOA was 300 ms (*M* = 431, *SE* = 25) rather than 150 ms (*M* = 462, *SE* = 25). All significant effects and interactions of the non-predictive Cue Condition model appear in Table 3.

**Table 3:** Significant effects from non-predictive Cue Condition model

Term	*F*	*df*	Residual df	*p*
Group	15.15	1	15	0.001
FSIQ	7.35	1	14.99	0.02
Validity	7.11	1	1518.01	0.008
SOA	78.86	1	1518.01	< 0.001
FSIQ:context	10.38	1	1518.01	0.001
Group:FSIQ:context	8.45	1	1518.01	0.004
Group:context:validity	7.48	1	1518.01	0.006
Group:context:validity:SOA	3.76	1	1518.01	0.05

**Fig. 3:**
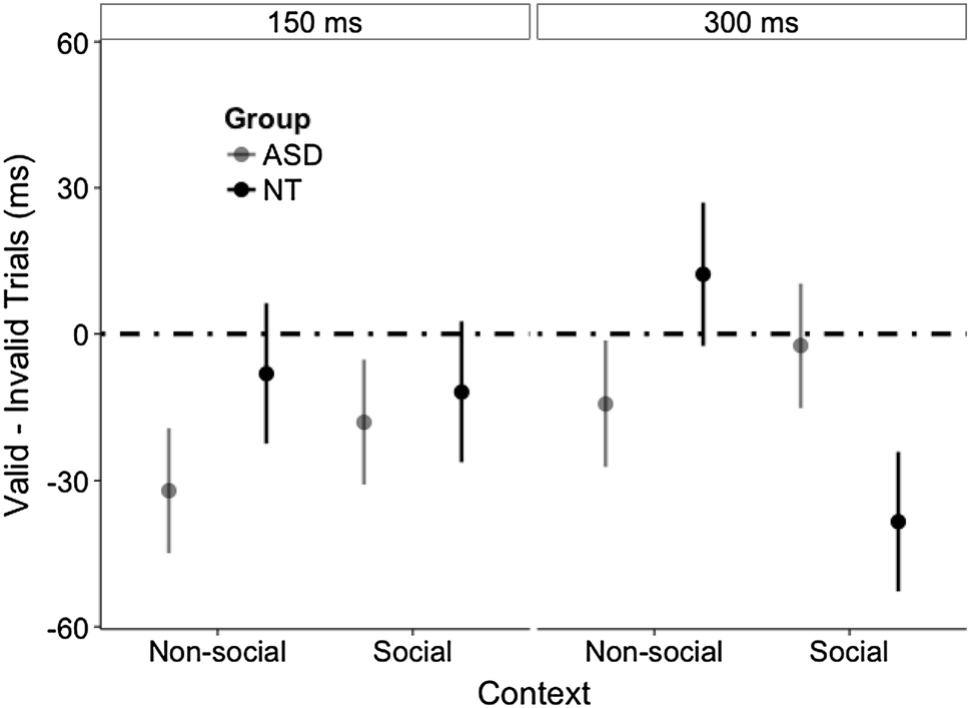
Cueing effects (valid–invalid trials) by group, context, and SOA in the non-predictive cue condition. Error bars represent 1 SEM

The non-predictive cue condition was used to test the second hypothesis. If the attentional response were automatic, we would expect to observe an RT advantage on valid trials (of cueing effect), despite non-predictive cue direction. In the non-predictive cue condition we used validity effects to explore the group × context × validity × SOA interaction (Fig. 3). In contrast to the ASD group, the NT group showed an automatic shift of attention, but only when the context was social and the SOA was 300 ms (*valid–invalid* = − 38 ms, *SE* = 14 ms), *t* (1518) = 3.5, *p* = .04, *d* = 0.18, all other *ps* > .97. The group × context × validity interaction was driven by this context × SOA interaction in the NT group: we observed a validity effect only for NT participants in the social context (*valid*–*invalid* = − 24 ms, *SE* = 15 ms), *t* (1518) = 3.5, *p* = .01, *d* = 0.18, all other *ps* > .14.

### Predictive Cue Condition

**Fig. 4:**
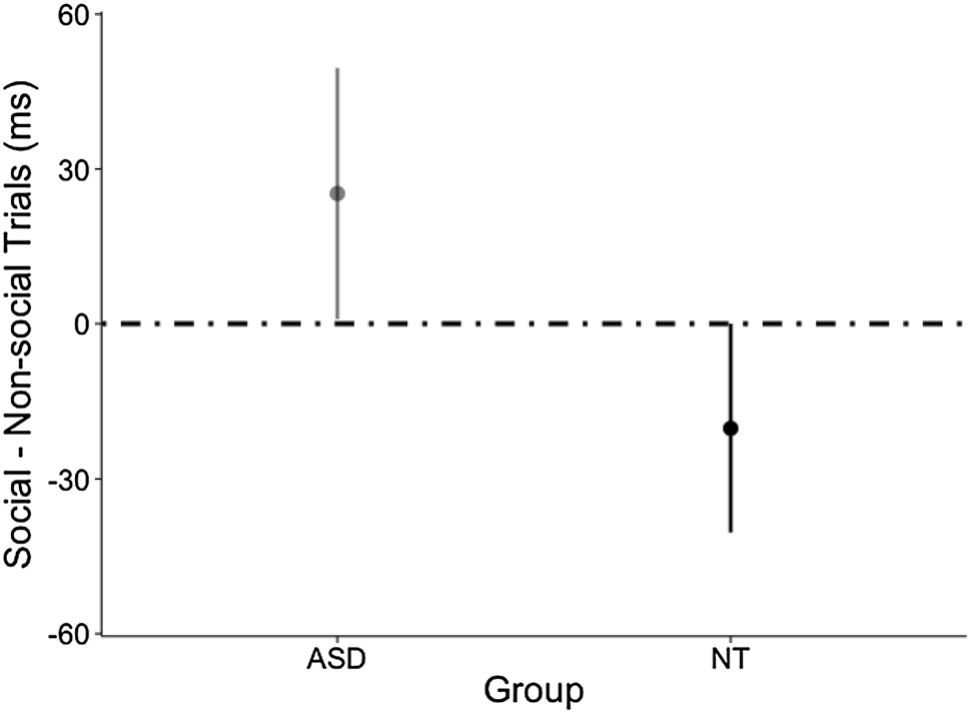
Reaction times on social minus non-social trials in the predictive cue condition by group. Error bars represent 1 SEM

The predictive cue condition was used to test the third hypothesis, that there would be group differences in the effect of social context. The significant group × context interaction (see Table 4) suggested that the effect of the social context was different across the two groups. The ASC group was slower on social trials, while NT participants were slower on non-social trials, as illustrated in Fig. 4.

**Table 4:** Significant effects from the predictive cue condition model

Term	*F*	*df*	Residual df	*p*
Validity	31.36	1	1583.03	< 0.001
SOA	59.87	1	1583.01	< 0.001
Group:context	11.63	1	1583.03	0.001
FSIQ:SOA	5.97	1	1583	0.015

We probed the group (ASC < NT) × context (social vs. non-social) interaction by subtracting non-social RTs from social RTs within each Group, thus measuring the effect of social context irrespective of validity or SOA (Fig. 4). The RT advantage for non-social (compared to social) conditions was not significant for the ASC group (*social–non-social* = 25 ms, *SE* = 24 ms), *t* (1583) = 1.8, *p* = .078, *d* = 0.09. Likewise, the RT advantage on social (compared to non-social) conditions was not significant for the NT group (*social–non-social* = − 20, *SE* = 20 ms t (1583) = 1.9, *p* = .056, *d* = 0.09. Nonetheless, a significant linear contrast comparing the differences between social and non-social trials across groups (NT[*social–non-social*]—ASC[*social–non-social*] contrast = − 45, *SE* = 14 ms), *t* (1583) = 3.2, *p* = .001, *d* = 0.16 revealed that social context had a different effect on NT participants than ASC participants. All significant effects and interactions of the Predictive Cue Condition model appear in Table 4.

**Fig. 5:**
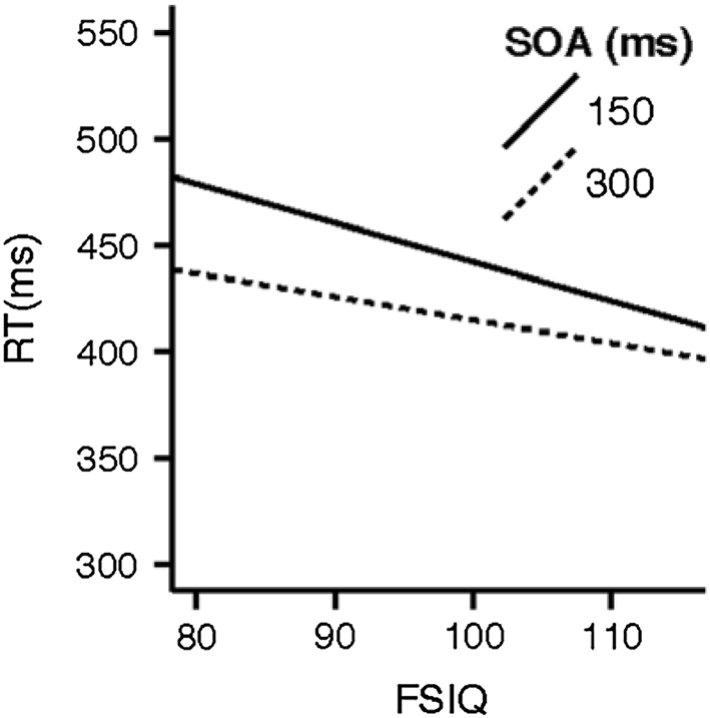
RT by FSIQ and SOA in the predictive cue condition

In the predictive cue condition, there was a significant interaction between FSIQ and SOA. People were faster for the 300 ms SOA than the 150 ms SOA. Higher FSIQ produced faster RTs for both SOAs, but the speed advantage with FSIQ was greater for the 150 ms SOAs than for the 300 ms SOAs. Figure 5 illustrates this interaction.

**Fig. 6:**
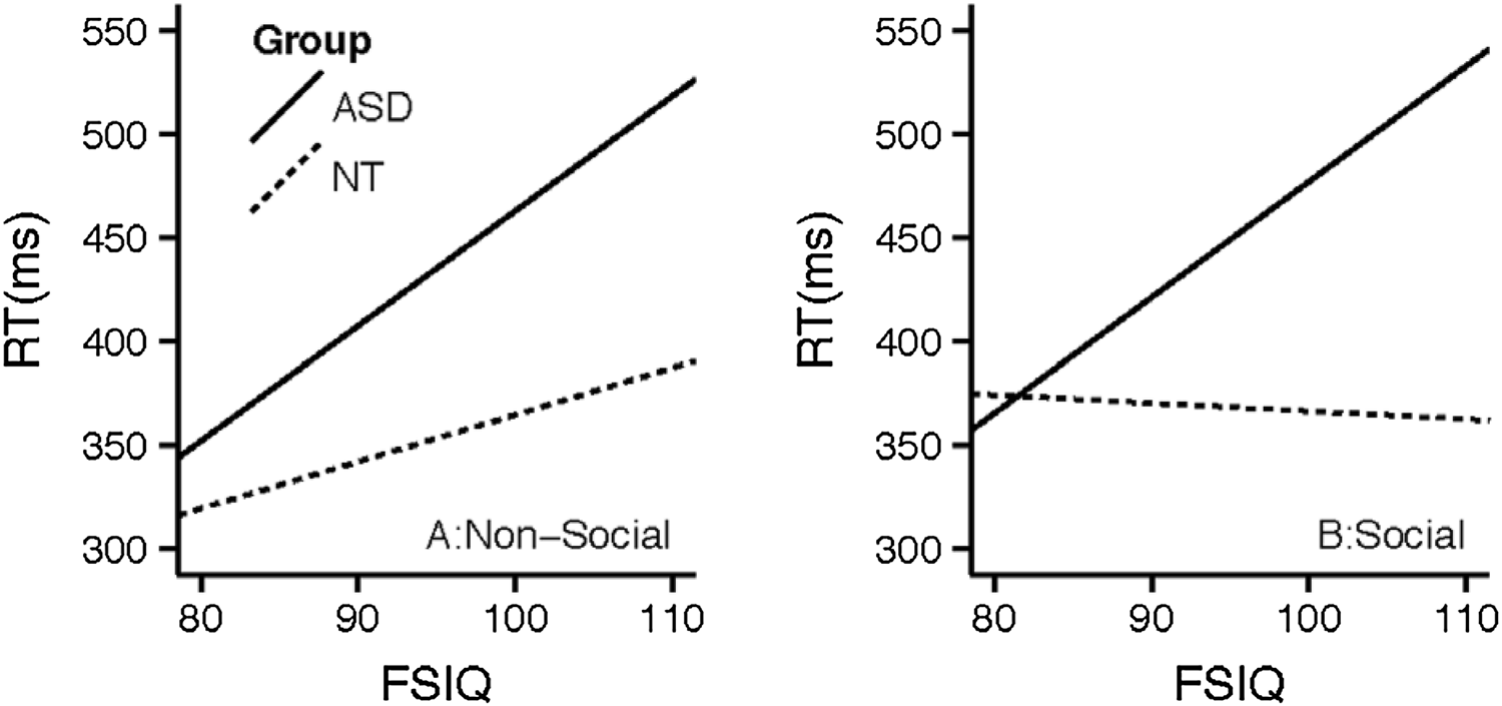
Reaction time by group, context and FSIQ, in the non-predictive cue condition

Further, in the non-predictive model, there was a group × FISQ × context interaction. An examination of that interaction revealed that higher FSIQ was associated with slower responses for individuals with ASD across contexts, but there was no association between FSIQ and RT for the NT group. Figure 6 illustrates this interaction.

## General Discussion

In this study we used a covert orienting paradigm to address three related questions about social orienting in social and non-social contexts among individuals with ASC and neurotypical development (NT): (1) Does sociality of the context affect orienting responses triggered by directional body cues? (2) Is such attentional orienting automatic? (3) Are there group differences in these effects?

The first hypothesis was that in the social context, more than in the non-social context, the central figure’s direction would trigger an attentional orienting response in the form of a larger validity effect (i.e., faster RTs for validly cued trials than invalidly cued trials). Even when cue direction was not predictive of target location, we found that NT participants showed a validity effect in the social context but not in the non-social context. This finding suggests that implied social interaction directed attention in NT participants. This social orienting was not found in ASC participants which we discuss below with respect to the third hypothesis.

The second hypothesis was that attentional cuing would be automatic. We tested this by examining validity effects in non-predictive cuing conditions and in shorter SOAs. Automatic cuing to social interaction was only observed in the NT group in the 300 ms SOA trials. This result differs from results observed with eye-gaze cueing in NT participants which have shown cueing effects at SOAs shorter than 150 ms (e.g., Green et al. 2013; Ristic et al. 2005) as well as for results observed with action image cues (same as the ones used in this study) by Gervais et al. (2010) which showed cueing effects at 100 ms SOAs. In both of these previous studies, the experimental conditions were different from this current study because the current study presented stimuli that included central cues with additional flanking images. Our results suggest that it may take longer to process both social and non-social contexts created by the combination of the flanking images with the central cue. In addition, our results suggest that the attentional response to meaningful body postures may not be as reflexive as to eye gaze, because it does not occur so rapidly that it is necessarily pre-conscious. Even if processing body postures were a more cognitively mediated process, it is automatic insofar as NT participants showed an attentional bias towards social interaction without prompting or direction from non-predictive cues. Further, in comparison to previous results (Gervais et al. 2010), embedding the central cue (the actor) in a context (social or otherwise) seemed to attenuate the orienting effect at the shortest SOA. It is possible that in our study the additional objects on the screen may have resulted in a more diffuse attentional focus (Castiello and Umiltà 1992).

The third hypothesis was that there would be social orienting differences between the those with and without ASC. Our results reveal such group differences. The ASC group did not show the automatic orienting response (hypothesis 1) with non-predictive social cues, while the NT group showed a validity effect in the social context. This test of effect of sociality on orienting responses was inspired by the social orienting view of autistic development. The social orienting view proposes that an early failure to orient to social information has social and cognitive developmental effects (Dawson et al. 2004; Mundy and Neal 2000). Our results are consistent with this view, insofar as participants with ASC do not show the of automatic orientation to social information as NT participants.

Importantly, the varying responses between NT and ASC groups in the social and non-social context strongly suggests that these differences are related to social processing. This interpretation aligns with the trends that Bayliss and Tipper (2005) observed using central eye-gaze cues. Among NT individuals, they found that those with low Autism Quotient (AQ) scores (i.e., who self-reported fewer autism-like traits) oriented faster when cued toward target images of intact rather than scrambled faces, while those with high AQ scores (who reported more autism-like traits) oriented faster when the target images were scrambled rather than intact faces. As in this study, both groups showed validity effects when cues were predictive of target location, regardless of the context. In other words, our study confirmed that individuals with ASC could perceive and use directional body cues when the cues were informative but did not use these cues when they were not predictive. Attentional cuing was likely driven by implicit learning of the contingency between the direction of the cue and location of the target, a form of learning that is believed to be intact in ASC (Brown et al. 2010; Nemeth et al. 2010).

Moreover, the impact of the sociality of the action was still measurable in the predictive condition for NT participants. Overall, their responses were faster in the presence of a social compared to a non-social target. When the display involved a social interaction and was predictive, NT participants had two cues directing their attention—the effects of contingent learning and a social orienting response. The availability of both cues may have accounted for their NT participants’ faster performance in the social versus non-social context, which included only one of those cues. Conversely, individuals with ASC responded faster with non-social compared to social interactions. This finding suggests that although individuals with ASC can orient to action implied by the human form, just as they can orient to eye-gaze cues (Pruett et al. 2011), implied social interaction may interfere with their responses. However, this finding contrasts with a meta-analysis reporting that those with ASC are more impaired with a non-social cue (an arrow) than with a social cue (eye direction) of attention (Landry and Parker 2013), and may be understood as a difference between the eye-gaze paradigm and the current implied action paradigm.

Although we manipulated similar-sized flanker stimuli to create relevant actions (i.e., it is functionally relevant to throw a ball at a tree target or to a person) with different social contexts, our results may be influenced by the fact that the two flankers differed in ways other than their social status. The social flankers are human figures that are inherently directional, while the non-social flankers are trees that are not directional. It is possible that participants’ attention was drawn towards social flankers *per se* because a social interaction is implied with any two individuals—they just vary in their interpretation (e.g., catching a ball versus observing a throw). Our design attempted to counteract this interpretation by including two human flankers facing opposite directions, cancelling out any directional draw of attention purely from the presence of another human, social stimuli. We found that the implied action of the cue directed attention more strongly to the flanker facing the cue. Nonetheless, in future studies it would be useful to compare performance in conditions that explicitly represent a functional interaction between the cue and the flankers but vary in their social nature, for example, including a throwing cue with flankers of a person ready to catch a ball or a receptacle positioned to catch the central thrower’s ball.

In addition, the trees had several top-down and bottom-up differences from the human figures that may influence the results. Indeed, other than being a potential target at which to throw a ball, it is possible that the lack of explicit functional relevance of the tree may play a part in the null findings in the non-predictive condition. Future experiments could test whether a bullseye as a target, or a ball-catching machine that provided a functional but non-social stimulus would enhance performance (controlling for the shape and size of the stimulus). Conversely, perhaps a non-interactive but still social flanker could be compared with the “catcher”, again contrasting function for sociality. For example, conditions with the human flanker facing outwards would create a non-social man stimulus could help to disambiguate the results. Finally, the social and non-social flankers could be equated for symmetrical direction (e.g. an asymmetric tree vs. the forward-facing man) could be employed in future studies.

Our study confirms previous findings that FSIQ scores are related to task performance for individuals with ASC but not NT individuals (Rutherford and Troje 2011; Black et al. 2009). As predicted, we found an association between higher FSIQ scores and faster RTs (Fig. 5). Further, FSIQ scores have social processing implications. This expected association was disrupted and even reversed in participants with ASC for non-predictive cue conditions (Fig. 6). Rutherford and Troje (2011) suggest that this ASC-specific pattern may represent an alternative, effortful strategy to solve a problem that is automatic or nearly automatic in NT individuals.

As a final point, as in any computer-based studies that imply social interaction (Senju et al. 2004; Kylliäinen and Hietanen 2004), we must be cautious in the generalization of the present results to real-world social interaction differences between NT and ASC individuals. The results of this study shed light on early attentional orienting from social cues because of its controlled set of stimuli and computer-based presentation. Although our findings correspond with reported real-world experiences, it would be difficult to speculate on how the study’s group differences in social orienting explain or contribute to the social cognitive irregularities seen in ASC in actual social interactions. Indeed, individuals with ASC are able to learn heuristics and alternative strategies that would be effective in conducting day-to-day social interactions, even if they take longer to execute. Nonetheless, this study contributes to our understanding of group differences in the influence of social context on early attentional cueing.

Figure 5 illustrates the predicted association between FSIQ and RT, since those with higher FSIQ scores would be expected to be faster. Figure 6 illustrates that this expected association was disrupted in participants with ASD when the cue was non-predictive. Under these conditions there is a robust reversal of this relationship. This finding was surprising, but it is not the first time that IQ has been found to be associated with performance in and ASD group while not in a control group. For example, perception of biological motion in point light displays is correlated with intelligence in ASD but not ND participants (Rutherford and Troje 2011). The authors of that study interpreted this association as representing an alternative, effortful strategy to solve a problem that is automatic or nearly automatic in ND individuals.

## Conclusions

This study provides several insights into what may be early processing atypicalities in social interactions for individuals with ASC. First, it suggests that social context enhances the cueing effect for NT individuals, producing a cueing effect even when the central cue is uninformative. In contrast, participants with ASC did not benefit from non-predictive social cues and were slower in social contexts. Nonetheless, they were able to benefit from predictive cues. Second, social orienting in a social context appears to be automatic for NT individuals in the sense that they do so without prompting. However, individuals with ASC provided no evidence of automatic social orienting. Hence the current results support the view that an illustrated representation of a human triadic interaction affects performance differentially in individuals with and without ASC in this attentional orienting task.
